# The complete chloroplast genome of *Verbascum chinense* (L.) Santapau

**DOI:** 10.1080/23802359.2020.1797556

**Published:** 2020-07-27

**Authors:** Yanxia He, Yuanyuan Ma, Zhengying Li, Peng Liu, Wangjun Yuan

**Affiliations:** a School of Life Sciences, Henan University, Kaifeng, China; b Institute of Chinese Materia Medica, Henan University, Kaifeng, China

**Keywords:** Chloroplast genome, *Verbascum chinense*, phylogenetic tree

## Abstract

*Verbascum chinense* is a perennial plant in the Scrophulariaceae family that is traditionally used as sedative, astringent, febrifuge, and for skin eruptions. Here, we determined the complete chloroplast(cp) genome sequence for *V. chinense* using genome skimming sequencing. The cp genome was 153,618 bp and consisted of a large single copy (LSC) region (84,834 bp), a small single copy (SSC) region (17,884 bp), and two inverted repeats (IRs) (25,450 bp). It encodes 114 unique genes, including 80 protein-coding genes, four rRNAs, and 30 tRNAs. Phylogenetic analysis indicates that *V. chinense* exhibits a closer relationship with *V. phoeniceum* rather than *Scrophularia.*

The genus *Verbascum* (Scrophulariaceae), commonly known as mullein, comprises about 360 species of flowering plants. *Verbascum* species possess a wide spectrum of biological activities; therefore, they have been used in traditional medicine for centuries (Georgiev et al. 2011). *Verbascum chinense* has conventionally been used as a sedative, an astringent, and a febrifuge as well as to treat skin eruptions (Kaur and Upadhyaya 2016). However, the phylogenetic position of *V. chinense* remains unclear, and there are limited studies on this species, particularly focusing on its genetics. The emergence of next-generation sequencing (NGS) technologies has enabled the rapid use of massive genomic data (He et al. 2017; Liu et al. 2017). The present study is the first to report the complete plastome of *V. chinense*. The plastome sequence is deposited in GenBank under the accession number MT610040.

The plant material of *V. chinense* was collected from Haroshi, Maharashtra, India (17°55′58.68″N, 73°37′36.67″E). The voucher specimen (#LP173132; collected by Pan Li) is stored at the Herbarium of Zhejiang University (HZU). Total genomic DNA was extracted from leaf tissue using Plant DNAzol Reagent (LifeFeng, Shanghai, China) according to the manufacturer’s protocol. High-quality DNA was sheared, and a paired-end library was prepared and sequenced on Illumina HiSeq X10 at Beijing Genomics Institute (BGI, Wuhan, China). The paired-end reads were trimmed and assembled into contigs using the CLC Genomics Workbench 9.5.2 (CLC Inc., Rarhus, Denmark). Next, the complete plastome of *V. chinense* was constructed using *Scrophularia henryi* (GenBank accession number: MF861203) as a reference sequence and annotated using Geneious R11 (Biomatters, Auckland, New Zealand), as described by Liu et al. (2018).

The complete plastome of *V. chinense* is 153,618 bp in length and includes a large single-copy (LSC) region of 84,834 bp, a small single-copy (SSC) region of 17,884 bp, and a pair of inverted repeat (IR) regions (IRa and IRb) of 25,450 bp. The plastome contains 114 unique genes, including 80 protein-coding, 30 tRNA, and four rRNA genes, as well as 20 duplicate genes in the IR regions. Six tRNA and eight protein-coding genes contain a single intron, whereas three genes (*rps12*, *clpP*, and *ycf3*) contain two introns. The overall GC content of the whole plastome is 38.0%, and the GC content of the LSC, SSC, and IR regions is 36.1%, 32.3%, and 43.2%, respectively.

A phylogenetic tree, including *V. chinense* and 28 other species in Lamiales, was constructed based on whole plastome sequences using the maximum likelihood (ML) method implemented in RAxML-HPC v8.1.11 on the CIPRES cluster (Stamatakis 2014); *Tiquilia plicata* was used as the outgroup. *V. chinense* formed a clade with *V. phoeniceum* and was sister to four *Scrophularia* species with a 100% bootstrap value (Figure 1).

**Figure 1.**
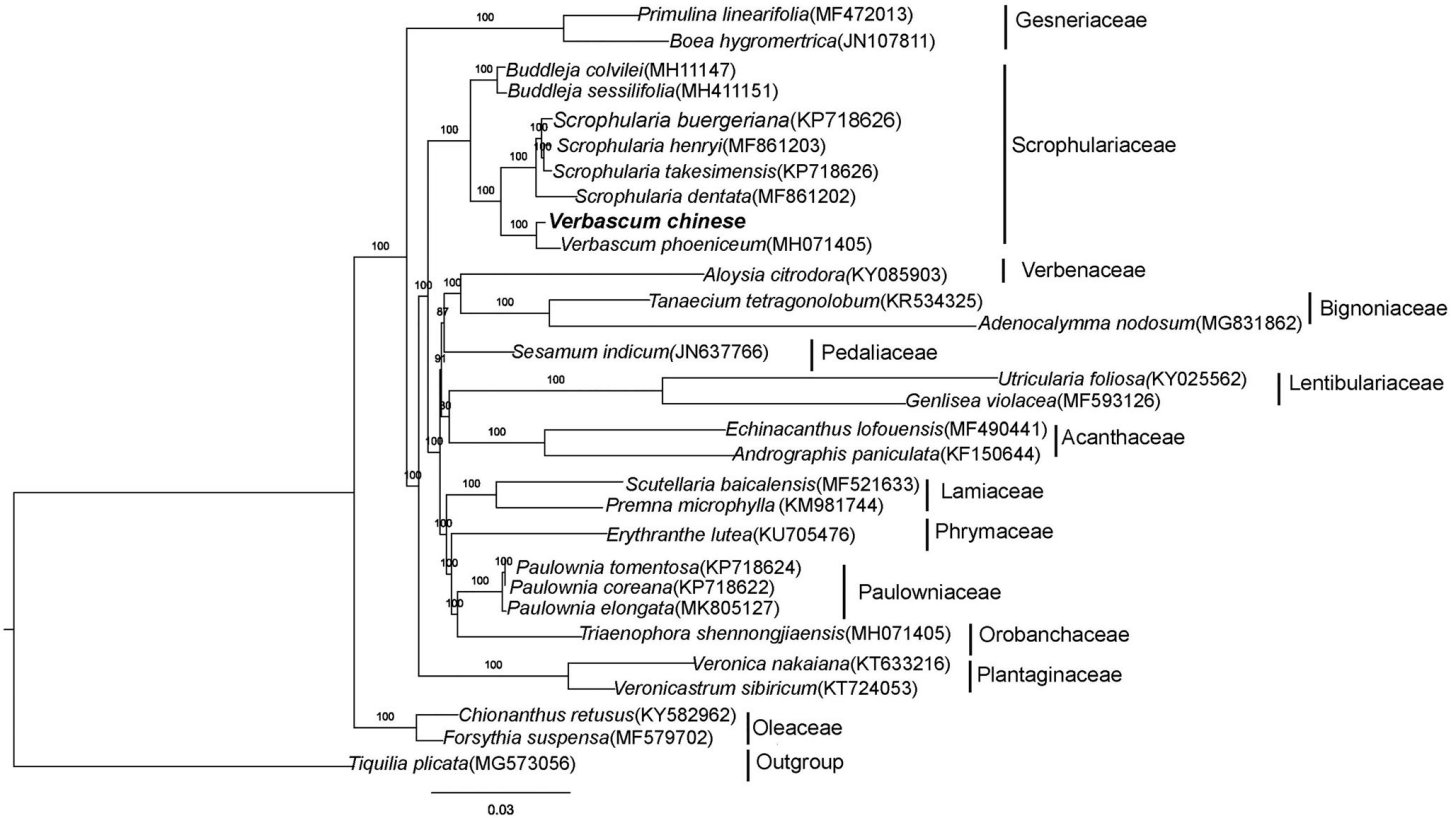
Phylogenetic relationships of Lamiales inferred based on whole chloroplast genome sequences. Number above each node indicates the ML bootstrap support values.

## Data Availability

The data that support the findings of this study are openly available in GenBank of NCBI at https://www.ncbi.nlm.nih.gov, reference number MT610040.
